# Increased Respiratory Modulation of Blood Pressure in Hypertensive Patients

**DOI:** 10.3389/fphys.2019.01111

**Published:** 2019-08-27

**Authors:** Lin Xie, Xiaohui Di, Fadong Zhao, Jie Yao, Zhiheng Liu, Chaomin Li, Binbin Liu, Xiaoni Wang, Jianbao Zhang

**Affiliations:** ^1^Key Laboratory of Biomedical Information Engineering of Education Ministry, Xi’an Jiaotong University, Xi’an, China; ^2^Department of Cardiology, No. 451 Hospital of Chinese People’s Liberation Army, Xi’an, China

**Keywords:** blood pressure, respiratory related variation, orthogonal subspace projection, mental stress, respiratory variability

## Abstract

**Objective:**

Although the important role of respiratory modulation of the cardiovascular system in the development of hypertension has been demonstrated in animal studies, little research has assessed this modulation in essential hypertensive patients. We aimed to explore whether respiratory-related variations in cardiovascular variables are changed in hypertensive patients and their potential relationships with the respiratory pattern.

**Methods:**

Respiration, ECG, and beat-to-beat blood pressure (BP) were simultaneously measured in 46 participants (24 hypertensive patients and 22 normotensive participants) during rest and a mental arithmetic task (MAT). Respiratory-triggered averaging and orthogonal subspace projection methods were used to assess the respiratory modulations of BP and heart rate (HR). Respiratory parameters including inspiratory time, expiratory time, respiratory rate and their variabilities were also characterized.

**Results:**

The inspiratory time, expiratory time, respiratory rate and their variabilities were not different between hypertensive and normotensives. Additionally, the modulation of HR by respiration was also similar between the two groups. Hypertensive patients exhibited an amplified respiratory modulation of systolic BP (SBP), as assessed from the amplitude of respiratory-related changes and the percentage of the power of respiratory-related variation, and also reflected from the temporal pattern of respiratory modulation of SBP. The exaggerated respiratory-related variation of SBP in hypertensive patients accounted for ≈23% of the total power of SBP, producing an absolute change of ≈4.5 mmHg in SBP. MAT was characterized by decreased inspiratory time and increased variabilities of expiratory time and respiratory rate with no changes in the amplitude of respiratory modulations.

**Conclusion:**

Hypertensive patients had excessive respiratory modulation of SBP, despite having similar respiratory pattern with normotensives. These findings highlight the importance of respiratory influence in BP variation and suggest that respiratory modulation of SBP may have prognostic information for cardiovascular events in hypertensive patients.

## Introduction

Hypertension remains a mounting threat worldwide despite the years of research and the significant advances in antihypertensive medication (Andreadis, 2016). Many systems, including the vasculature, the nervous system, the kidney, and the respiratory system, along with their interactions contribute to the blood pressure (BP) homeostasis, and the dysregulation of BP thus may result from the failure of one or more of these systems (Coffman, 2011). Accumulating evidence has suggested a critical role of the respiratory system in the development and maintenance of hypertension (Joseph et al., 2005; Oneda et al., 2010; Briant et al., 2015; Moraes et al., 2016). The respiratory system, which interacts closely with the cardiovascular system via both central interaction between cardiovascular and respiratory neural circuits in the brainstem and modulatory feedback signals from a range of peripheral reflexes, contributes significantly to the regulation of BP. Recent animal studies have demonstrated that respiratory modulation of BP was dramatically amplified in spontaneously hypertensive rats (Simms et al., 2009; Menuet et al., 2017) and in other experimental models of hypertension (Moraes et al., 2012). Moreover, this exaggerated respiratory modulation is related not only to the maintenance but also to the initiation of hypertension (Simms et al., 2009). In juvenile spontaneously hypertensive rats, selectively eliminating specific neurons in the rostral ventrolateral medulla (RVLM), which reduced the amplitude of respiratory variation in BP, decreased adult BP by ∼20 mmHg. Furthermore, the amplitude of respiratory modulation and mean BP (MBP) exhibited a strong relationship throughout the development of high BP in those rats (Menuet et al., 2017). In another experimental model of hypertension in which rats were submitted to chronic intermittent hypoxia, marked increases in the respiratory modulations of sympathetic activity and BP were observed with augmented expiratory activity, suggesting the alterations in baseline respiratory pattern may contribute to changes in cardiorespiratory interaction as well as the maintenance of BP homeostasis (Malpas, 2010; Moraes et al., 2012).

Despite the findings of the significant contribution of respiratory modulation of the cardiovascular system to the development of hypertension in animal models, there is a paucity of information about this modulation in hypertensive patients. One study (Fatouleh and Macefield, 2011) focusing on the respiratory-sympathetic coupling in hypertensive patients showed inconsistent results compared with animal studies. Hypertensive patients and healthy control subjects exhibited similar cardiac and respiratory-related muscle sympathetic activity at rest (Fatouleh and Macefield, 2011). However, the respiratory-related variation in BP was not assessed. Furthermore, the responses of vasoconstriction and BP to sympathetic activation can be exaggerated even the resting sympathetic activity remains normal (Fink, 2018; Vranish et al., 2018). Given the important role of respiratory modulation of BP in essential hypertension as demonstrated in animal experiments, we were interested to test if it is also exaggerated in hypertensive patients and whether this might be related to a potential change of resting respiratory pattern. Furthermore, both cardiovascular and respiratory systems, as well as their interaction, exhibit significant changes under mental stress. The investigation of respiratory-related variations in cardiovascular variables during mental challenge could provide additional insights into the potential difference in cardiorespiratory coupling between healthy controls and hypertensive patients. In this study, we calculated the absolute and normalized magnitudes of respiratory modulation of BP as well as the percentage of the power of respiratory-related variation in BP in normotensive controls and hypertensive patients during rest and mental challenge. Respiratory parameters including inspiratory time, expiratory time and respiratory rate and their variabilities were also examined. We hypothesized that hypertensive patients would exhibit an increased respiratory modulation of BP compared to normotensives, and mental stress would alter the respiratory modulation of cardiovascular variables due to the changes in cardiorespiratory parameters.

## Materials and Methods

### Participants

A total of 50 participants, comprising 25 hypertensive patients and 25 normotensive controls between 40 and 60 years of age were recruited. All participants were screened by medical history, 12-lead ECG, and routine physical examination in the department of cardiology of No. 451 Hospital of Chinese People’s Liberation Army. The measurement of BP was taken at both arms with participants rested for 10 min at sitting position, using the auscultatory method with a mercury sphygmomanometer. The average of BP measured from both arms were documented. BP was measured at two clinic visits. Participants with systolic BP (SBP) < 120 mmHg and diastolic BP (DBP) < 80 mmHg during both visits were classified as normotensives, and hypertension was defined as SBP ≥ 140 mmHg or DBP ≥ 90 mmHg (Chobanian et al., 2003). Exclusion criteria included secondary hypertension, borderline/prehypertension (office BP of 130/80 to 140/90 mm Hg), diabetes, cardiac dysrhythmias, neurological disease, peripheral vascular disease, respiratory disease and body mass index (BMI) > 30. Recordings from 4 participants (1 hypertensive and 3 normotensive participants) were excluded because of the poor signal quality, and the final dataset was therefore obtained from 46 participants. Fifteen participants were women (9 in the hypertensive group and 6 in the control group) and 12 (80%) of them were postmenopausal. The type and number of patients taking antihypertensive medication in hypertensive groups were angiotensin-converting enzyme inhibitors (6), angiotensin-II receptor blockers (3), calcium channel blocker (7), diuretics (4), and ß-blockers (1). None of the participants in the control group was on medications at the time of this study. This study was approved by the Ethical Committee of Xi’an Jiaotong University and all participants gave written informed consent to participate.

### Experimental Protocol

Before the experimental visit, participants were asked to refrain from caffeine, alcohol, and vigorous exercise for at least 24 h. All experiments were performed in an environment-controlled room (soundproof, temperature: ≈22°C). Respiratory movements were measured by a strain-gauge pressure belt that placed stably around the chest (MP150, BIOPAC Systems Inc.). A 3-lead ECG (Lead II configuration) was also collected by the MP150 system. Beat-to-beat BP was measured through a photoplethysmograph finger cuff from Finometer (Finapres Medical Systems, Amsterdam, Netherlands). The Finometer cuff was wrapped around the middle finger of the participant’s non-dominant hand and referenced to the heart level. The raw pulsatile pressure data from the Finometer was input to the MP150 system in order to simultaneously record ECG, respiratory and BP signals. After the instrumentation and experimental setup, participants rested comfortably in a supine position for 20 min. Respiratory and cardiovascular signals were then recorded continuously throughout a 5-min baseline period followed by a 5-min mental arithmetic test (MAT). MAT involved continuous subtraction of seven from a four-digit number, and the participants were instructed to calculate as fast and accurately as possible. Throughout the procedure, participants lay comfortably and breathed spontaneously.

### Data Analysis

Offline analysis of ECG, BP and respiratory signals was carried out by scripts written in Matlab (The MathWorks, Natick, MA, United States). ECG signal was first bandpass filtered (5–15 Hz), and then Pan-Tompkins algorithm was used to detect R peaks (Pan and Tompkins, 1985). Beat-to-beat heart rate (HR) was derived from the series of R–R interval. The beat-to-beat SBP and DBP were extracted from the BP waveform. MBP was calculated as (*SBP* + 2*DBP*)/3. The time series of HR, SBP, DBP, and MBP were interpolated and resampled at 2 Hz for further analysis. Stroke volume (SV) was estimated from the pulse waveform using the Modelflow method embedded in Finometer (Jansen et al., 2001). Cardiac output (CO) is the product of SV and HR, and systemic vascular resistance (SVR) is the quotient of MBP and CO. Respiratory signal was smoothed by a Savitzky–Golay filter with an order of 1 (Kralemann et al., 2013), and the peaks and troughs were extracted to calculate respiratory parameters, including inspiratory time, expiratory time, and respiratory rate. The variabilities of these respiratory parameters were estimated using the root mean square of successive differences over consecutive breaths (Anderson et al., 2008).

### Respiratory Modulation of HR and BP

The respiratory-related changes in HR and BP were examined using the event-triggered averaging method. The inspiratory peaks were set as the triggering events in this study. For each participant, HR signal was segmented into epochs from his/her mean inspiratory time before to his/her mean expiratory time after the inspiratory peaks. These epochs were then averaged to generate the respiratory-related HR wave. The averaging process could dampen the irrelevant variabilities in HR and generate a distilled representation of the respiratory-related HR wave. The quantification of respiratory modulation on HR was then performed by measuring the difference between the maxima and minima of the averaged HR wave. Both absolute (ΔHR: maxima-minima) and normalized changes (Norm_ΔHR: maxima-minima/maxima) of HR caused by respiration were calculated in this study. Identical respiratory-triggered averaging procedures were performed for SBP, DBP, and MBP time series to assess respiratory modulation of blood pressure.

The respiratory-related HR and BP waves derived from the event-triggered averaging method could also be used to identify the temporal relationship between respiration and cardiovascular variables. The individual duration of this HR (or BP) wave varied among participants due to their different durations of inspiration and expiration, which would make the direct inter-subject averaging impossible. In order to characterize the temporal respiratory modulation of HR (or BP) at the group level, a novel method based on respiratory phase was used in this study. For each participant, the HR (or BP) wave was resampled and interpolated with five-points from the onset of the inspiration to the inspiratory peak and five-points from the inspiratory peak to the end of the expiration using cubic spline interpolation. This approach was well suited to align the respiratory-related segments of cardiovascular variables within one respiratory period for multi-subject analysis, allowing the characterization of the temporal respiratory modulation at group level. After the interpolation, the HR (or BP) values were subtracted by the value of HR (or BP) at the inspiratory peak to represent a relative change during one respiratory cycle.

### Orthogonal Subspace Projection

One strategy to distill the respiratory-related variability from the total variability of cardiovascular signals is to decompose the signals into two components, namely the respiratory-related component and component related to other rhythms. Many methods have been proposed in the field of biomedical signal processing for this decomposition (Choi and Gutierrez-Osuna, 2011; Widjaja et al., 2014). A recent methodological evaluation study showed that the orthogonal subspace projection yielded better performance regarding the separation of the respiratory influence from the tachogram (Widjaja et al., 2014). In this regard, the orthogonal subspace projection was used to extract the respiratory-related components in cardiovascular variables in the present study. The projection of HR (or BP) signal to the respiratory subspace could decompose HR (or BP) signal into two parts: the part in the subspace, which corresponds to the respiratory-related component, and the part that is orthogonal to the subspace. Therefore, the power of the respiratory-related component of HR (or BP) signal could be calculated and serves as an index of respiratory influence on HR (or BP). Accordingly, the first step of the algorithm is to construct the respiratory subspace (V), which was formed by wavelet coefficients and their delayed versions of respiratory signal (Caicedo et al., 2012; Widjaja et al., 2014). The level of the wavelet decomposition was 5 and the delay was set at 3 s in this study. After the construction of the respiratory subspace, the projection matrix P was calculated:

$$P=V\,{\left(V^{T}\,V\right)}^{-1}\,V$$

The projected signal of HR (*HR*_*resp*_) and BP (*BP*_*resp*_) were then given by:

$$H\,R_{r\,e\,s\,p}=P\cdot H\,R$$

$$B\,P_{r\,e\,s\,p}=P\cdot B\,P$$

Finally, the quantification of the respiratory influence on HR (or BP) was performed by computing the ratio between the power of the respiratory-related component and the total power of HR (or BP) signal (Varon et al., 2015), expressed as a percentage:

$$PowerofHR_{r\,e\,s\,p}\left(\%\right)=\frac{H\,R_{r\,e\,s\,p}^{T}\,H\,R_{r\,e\,s\,p}}{H\,R^{T}\,H\,R}$$

$$PowerofBP_{r\,e\,s\,p}\left(\%\right)=\frac{B\,P_{r\,e\,s\,p}^{T}\,B\,P_{r\,e\,s\,p}}{B\,P^{T}\,B\,P}$$

### Statistical Analysis

Participants’ characteristics were summarized as means for continuous variables and percentages for categorical variables. The primary outcome of the present study was a difference in respiratory modulation of BP between normotensive and hypertensive groups and we estimated a 50% difference between the two groups for the power calculation. Based on previous research, the respiratory modulation of SBP would be 2.5 mmHg in the control group (Menuet et al., 2017). Assuming an SD of 1.2 mmHg, 80% power, and a 2-sided αlevel of 0.05, the necessary sample size would be 17 participants per group. A χ^2^ analysis was performed for comparisons of the categorical data. The distributions of continuous variables were examined by the Shapiro–Wilk test. For variables with normal distribution, between-group comparisons were carried out by the independent *t*-test and within-group comparisons between baseline and MAT conditions were done by paired *t*-test. For variables with skewed distributions, Mann–Whitney test and 1-sample Wilcoxon test were performed for between and within group comparison, respectively. A mixed-model ANOVA with respiratory phase and condition (rest and MAT) as within-subject factors and group (normotensive and hypertensive) as the between-subjects factor, was used to analyze the temporal regulation of respiration on HR and BP. Data are expressed as means ± SEM, and α level was set at 0.05.

## Results

### Cardiovascular Parameters

Table 1 presents the characteristics of the participants. The normotensive and hypertensive groups did not differ significantly in their age, height, weight, BMI, gender, and smoking status. Cardiovascular variables of the two groups during rest and MAT are listed in Table 2. At rest, the mean value of SBP/DBP was 115.41 ± 1.79/70.14 ± 1.20 mmHg for the normotensive group and 153.50 ± 2.88/88.35 ± 1.88 mmHg for the hypertensive group. Compared with normotensive participants, hypertensive patients had significantly higher SVR at rest and MAT conditions, whereas HR, SV, and CO were not different between the two groups. Compared to rest, MAT elicited significant increases in BP, HR, and CO in normotensive and hypertensive participants.

**TABLE 1:** Participant characteristics.

	**Normotensives**	**Hypertensives**
Age, years	49 ± 1	51 ± 1
Female,%	27%	37%
Height, cm	169 ± 2	167 ± 1
Weight, kg	71 ± 2	71 ± 2
BMI, kg/m^2^	24 ± 1	25 ± 1
Smokers,%	36%	45%

*Values are means ± SEM. BMI, body mass index.*

**TABLE 2:** Cardiovascular parameters at rest and MAT.

**Variable**		**Normotensives**	****Hypertensives****
SBP, mmHg	Rest	115.41 ± 1.79	153.50 ± 2.88^†^
	MAT	122.30 ± 1.94^∗^	160.64 ± 3.43^∗†^
DBP, mmHg	Rest	70.14 ± 1.20	88.35 ± 1.88^†^
	MAT	73.69 ± 1.34^∗^	91.54 ± 2.17^∗^^†^
MBP, mmHg	Rest	85.23 ± 1.31	110.07 ± 2.04^†^
	MAT	89.89 ± 1.46^∗^	114.57 ± 2.42^∗^^†^
HR, beats/min	Rest	71.70 ± 2.46	72.11 ± 2.09
	MAT	73.59 ± 2.54^∗^	74.35 ± 2.14^∗^
SV, ml	Rest	78.33 ± 3.76	73.06 ± 4.21
	MAT	79.09 ± 3.29	73.57 ± 3.85
CO, l/min	Rest	5.45 ± 0.22	5.25 ± 0.30
	MAT	5.66 ± 0.20^∗^	5.39 ± 0.32^∗^
SVR, dyn⋅s⋅cm^–5^	Rest	1360.04 ± 56.31	1900.73 ± 127.33^†^
	MAT	1379.24 ± 50.41	1937.94 ± 141.79^†^

*Values are means ± SEM. MAT, mental arithmetic task; HR, heart rate; SBP, systolic blood pressure; DBP, diastolic blood pressure; MBP, mean blood pressure; SV, stroke volume; CO, cardiac output; SVR, systemic vascular resistance. ^∗^*P* < 0.05 vs. rest. ^†^*P* < 0.05 vs. normotensives.*

### Respiratory Parameters

**FIGURE 1:**
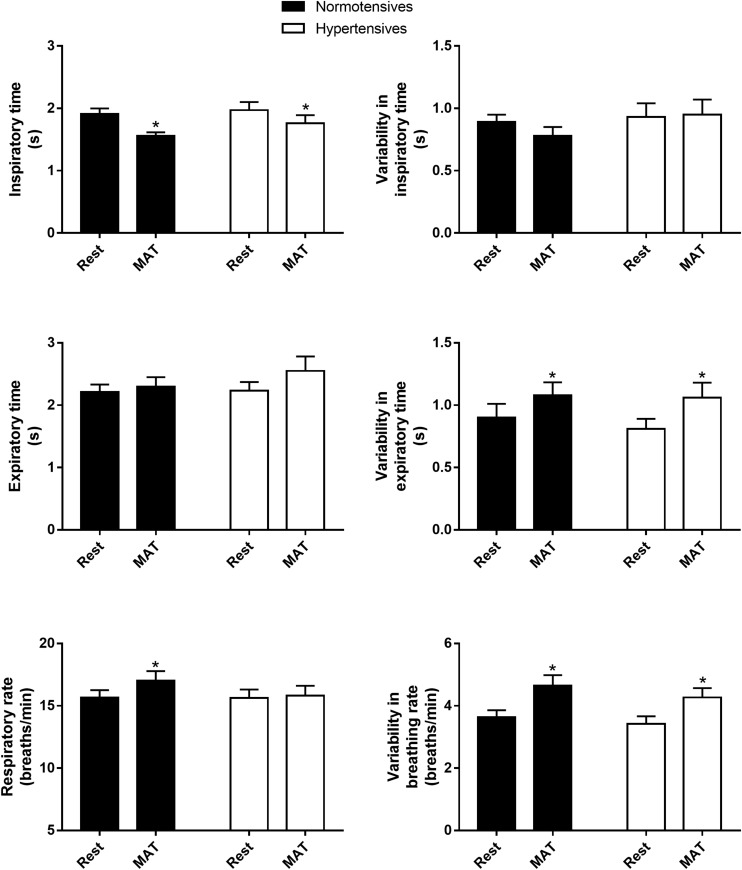
The inspiratory time, expiratory time, respiratory rate and their variabilities in normotensive and hypertensive groups at rest and MAT. ^∗^*P* < 0.05 vs. rest.

As shown in Figure 1, inspiratory time, expiratory time, respiratory rate and their variabilities were similar between normotensives and hypertensives at rest and MAT. MAT led to a significant decrease in inspiratory time and an increase in respiratory rate in the normotensive group. Similarly, the hypertensive group also showed a decrease in inspiratory time during MAT, but the expiratory time tended to increase in this group (2.23 ± 0.14 vs. 2.55 ± 0.23 s, *P* = 0.067), and as a result, respiratory rate did not change significantly in hypertensives during MAT. In addition, the variabilities of expiratory time (controls: 0.90 ± 0.11 versus 1.08 ± 0.10, *P* = 0.029; hypertensives: 0.81 ± 0.08 vs. 1.06 ± 0.12, *P* = 0.024) and respiratory rate (controls: 3.63 ± 0.23 vs. 4.65 ± 0.33, *P* = 0.003; hypertensives: 3.42 ± 0.24 vs. 4.27 ± 0.30, *P* = 0.007) were significantly increased during MAT in both groups.

### Respiratory Modulation

**FIGURE 2:**
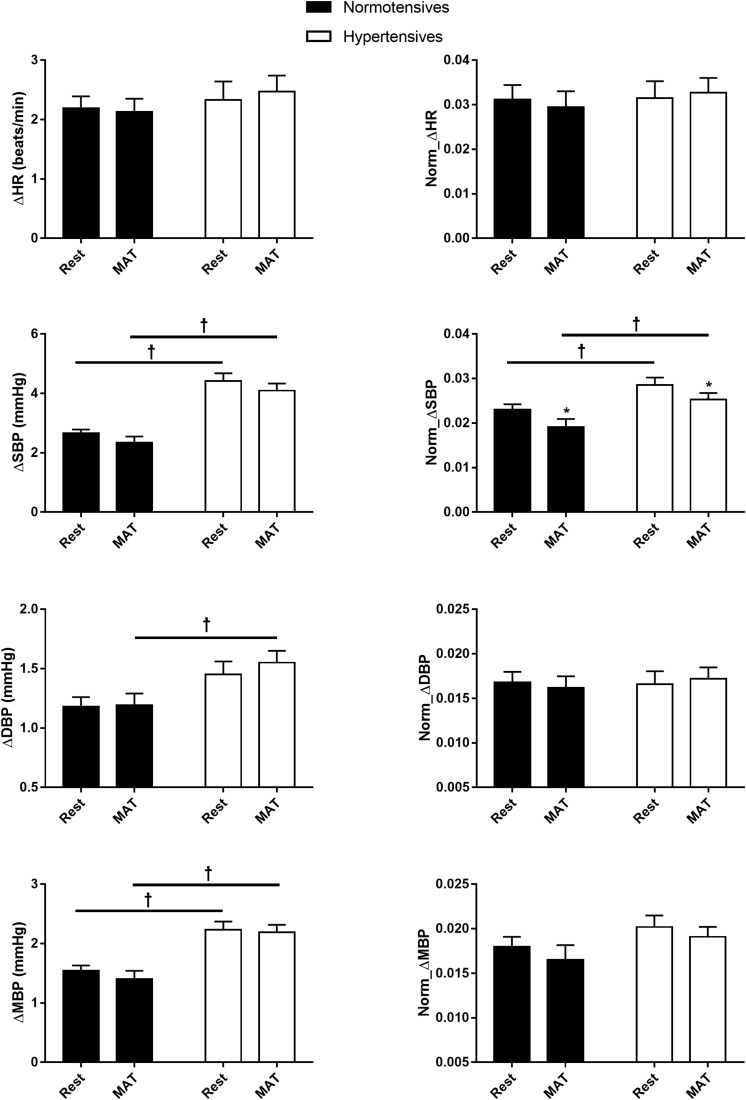
The respiratory-related changes in HR, SBP, DBP, and MBP in normotensive and hypertensive groups at rest and MAT. The changes were presented in absolute (ΔHR, ΔSBP, ΔDBP, ΔMBP) and normalized values (Norm_ΔHR, Norm_ΔSBP, Norm_ΔDBP, Norm_ΔMBP). ^∗^*P* < 0.05 vs. rest. ^†^*P* < 0.05 vs. normotensives.

**FIGURE 3:**
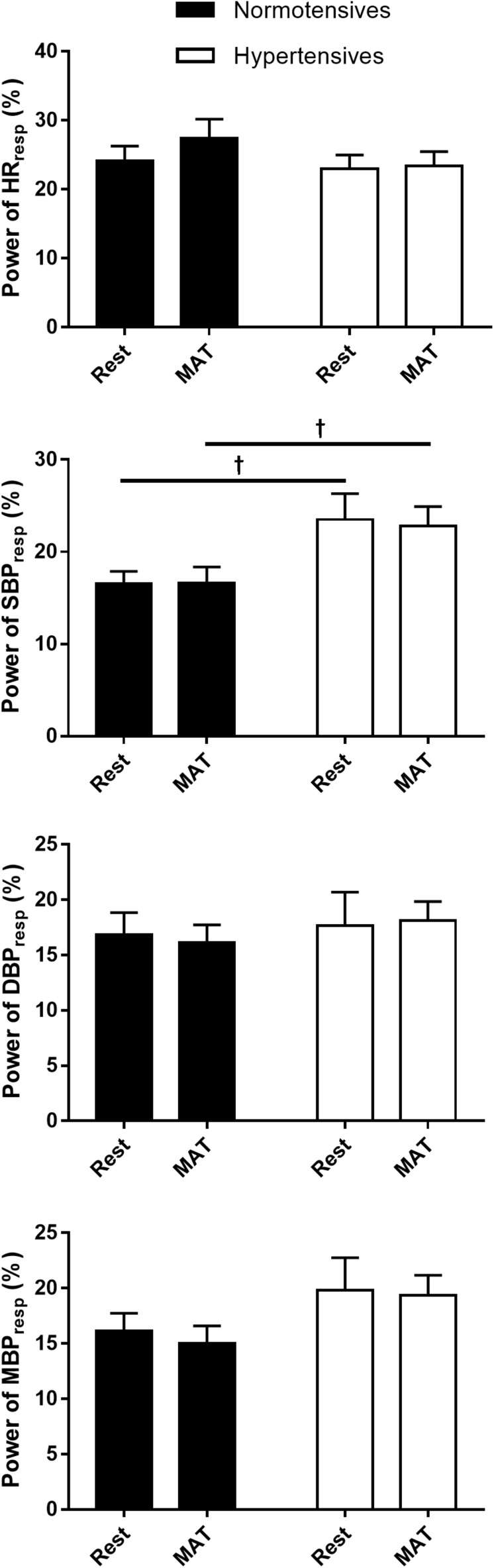
The percentage of the power of HR, SBP, DBP, and MBP that regulated by respiration in normotensive and hypertensive groups at rest and MAT. ^†^*P* < 0.05 vs. normotensives.

Figure 2 shows the absolute and normalized respiratory-related changes in HR and BP computed by event-triggered averaging method. The percentages of the powers of the respiratory-related variations in HR (*HR*_*RESP*_), SBP (*SBP*_*RESP*_), DBP (*DBP*_*RESP*_), MBP (*MBP*_*RESP*_), determined by orthogonal subspace projection, are presented in Figure 3. The results from the two methods consistently showed an exaggerated resting respiratory modulation of SBP in hypertensive patients (ΔSBP: normotensives: 2.65 ± 0.13 mmHg, hypertensives: 4.41 ± 0.27 mmHg, *P* < 0.001; percentage of power in total variation: normotensives: 16.54 ± 1.36%, hypertensives: 23.52 ± 2.78%, *P* = 0.034). On the other hand, the two groups exhibited similar respiratory modulation of HR at rest (ΔHR: normotensives: 2.19 ± 0.20 beats/min, hypertensives: 2.33 ± 0.31 beats/min; percentage of power in total variation: normotensives: 24.16 ± 2.31%, hypertensives: 23.02 ± 1.96%). Additionally, resting modulation of DBP by respiration was also comparable between the two groups. The resting ΔMBP was significantly higher in hypertensive patients, but this difference became non-significant after the normalization. Similar to the baseline condition, hypertensives and normotensives showed comparable ΔHR, Norm_ΔHR, Norm_ΔDBP, and Norm_ΔMBP during MAT, whereas ΔSBP, ΔDBP, ΔMBP, and Norm_ΔSBP were significantly larger in hypertensive patients.

**FIGURE 4:**
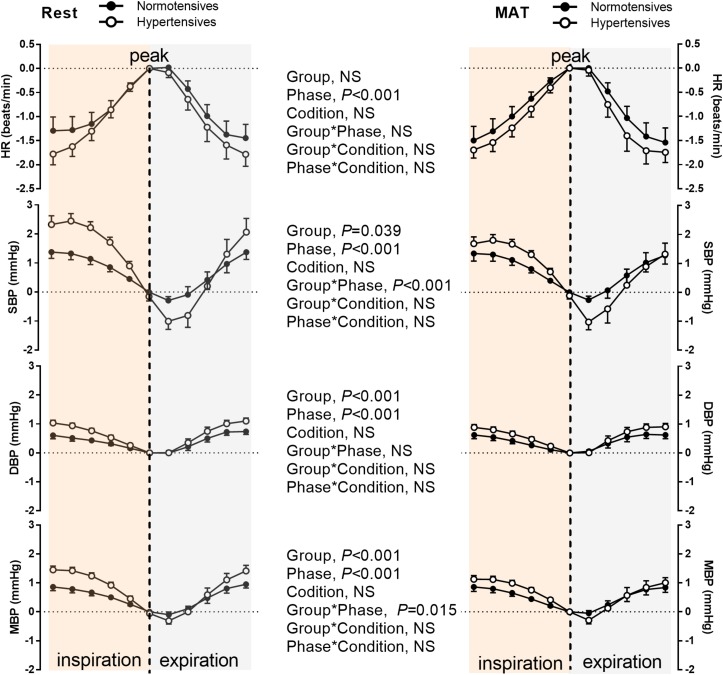
The HR, SBP, DBP, and MBP during one respiratory cycle in normotensive and hypertensive groups at rest and MAT. Note that the values of HR, SBP, DBP, and MBP were subtracted by the reference values, which was the value of corresponding signal at the inspiratory peak. The main effects of group, respiratory phase, and condition as well as their interactions were analyzed using a mixed-model ANOVA and the results are shown in the **middle panel**. NS indicates not significant.

Figure 4 depicts the group averaged variations in HR, SBP, DBP, and MBP within one respiratory period. As can be seen, HR and BP were significantly modulated by respiration (*P* < 0.001, ANOVA main effect of respiratory phase). HR increased during inspiration, and reached the maximum at the end of inspiration, then decreased during expiration. BP decreased during most of the inspiratory period and expiration was associated with gradually rising BP as previously reported (Dornhorst et al., 1952). The changes in HR during one respiratory cycle were similar between normotensive and hypertensive groups (No significant main effect of group or group × respiratory phase interaction). Conversely, a significant main effect of group was observed in the respiratory modulation of SBP, DBP, and MBP. There was a significant interaction between the groups and respiratory phase on the changes in SBP (*P* < 0.001) and MBP (*P* = 0.015), suggesting the degrees of inspiratory elevation and post-inspiratory attenuation of them were different between the two groups. The main effect comparing the rest and MAT conditions in the respiratory modulation of HR and BP were not significant.

## Discussion

Our novel finding is that the respiratory modulation of SBP is exaggerated in hypertensive patients compared with age, sex, and BMI matched normotensive subjects. Additionally, different from our initial hypothesis, the changes in respiratory pattern and cardiovascular variables during MAT did not affect the respiratory-related variations in cardiovascular variables. An absolute respiratory-related change of ≈4.5 mmHg in SBP was observed in hypertensive patients with normal resting respiratory pattern, and the respiratory-related variation accounted for ≈23% of the total variability of SBP in those patients. These findings highlight the importance of respiratory influence on BP variation. Given that increased BP variability is significantly associated with cardiovascular and mortality outcomes (Parati et al., 2013; Mehlum et al., 2018), the exaggerated SBP variation modulated by respiratory activity in hypertensive patients may have considerable clinical relevance and prognostic value.

### Respiratory Modulation at Rest

It has long been known that the cardiovascular and respiratory systems are closely linked, and the respiratory modulations of HR and BP are two important manifestations of this physiological coupling (Attinger, 1957; Machado et al., 2017). Although the mechanisms underlying the respiratory influence on HR and BP are still elusive, it was proved that they were associated with different aspects of respiratory modulation of autonomic activity. Given the sympathetic effects on HR are too slow to follow the respiratory frequency, the respiratory modulation of HR is mainly mediated by vagal outflow (Warner and Cox, 1962; Farmer et al., 2016). Our data showed that respiratory modulation of HR was not different between the normotensive and hypertensive groups, indicating the respiratory modulation of vagal outflow was similar between the two groups. On the other hand, variations in the sympathetic nerve discharges with respiratory rhythm, which elicit phasic vasoconstriction within the vasculature, contribute significantly to the respiratory modulation of BP (Briant et al., 2015; Shantsila et al., 2015). Sympathetic nerve activity usually exhibits a pattern of respiratory modulation with an excitatory response during inspiratory and/or post-inspiratory periods (Häbler et al., 1994; Molkov et al., 2014). Conversely, our observation of the temporal relationship between blood pressure and respiration shows a decrease in blood pressure during inspiratory and post-inspiratory phases (Figure 4). This inverse relationship between sympathetic activity and blood pressure may largely due to the baroreflex function (Charkoudian and Rabbitts, 2009). During inspiration, the decreased intrathoracic pressure causes an increase in blood flow to the right side of the heart. Furthermore, the capacity of the pulmonary arteries also increased during inspiration, which facilitates blood pooling into the lungs. These changes decrease the filling of the left side of the heart and compromise left cardiac stroke volume, and consequently lead to a decrease in blood pressure during inspiration (Elstad et al., 2018). The decrease in blood pressure is sensed by the arterial baroreceptors and elicits an increase in sympathetic nerve activity. Similarly, the increased blood pressure during expiration inhibits sympathetic activity via the baroreflex. These baroreflex responses contribute to a reciprocal relationship between sympathetic nerve activity and blood pressure during respiration. Importantly, our study demonstrated that the respiratory modulation of SBP significantly increased in hypertensive patients, as assessed from both ΔSBP, Norm_ΔSBP, power of SBPresp, and the temporal variation of SBP within one respiratory cycle. This observation is in line with experimental studies using animal models of hypertension (Simms et al., 2010; Moraes et al., 2014; Menuet et al., 2017). Our initial hypothesis was that the exaggerated respiratory modulation of BP may be associated with changes in respiratory activity in hypertensive patients. However, we found no significant changes in inspiratory duration, expiratory duration, respiratory rate and the breathing variability in hypertensive patients compared to normotensives, indicating no resting respiratory dysfunction in hypertensive patients. A study by Anderson et al. reported an increased variability of respiratory rate in women with higher BP (Anderson et al., 2008). The respiration during night time sleeping was included in their analysis, whereas our study only involved daytime recordings, which may explain the inconsistent observations.

The precise mechanism of the exaggerated respiratory modulation of BP in hypertensive patients remains unclear, but altered chemoreflex function in hypertension could be a potential major mechanism (Zoccal and Machado, 2011; McBryde et al., 2013). Enhanced peripheral chemoreflex sensitivity has been shown in both animal and human studies of hypertension (Schultz et al., 2007; McBryde et al., 2013). In spontaneously hypertensive rats, the elevated respiratory drive, which may be largely attributed to the potentiated chemoreflex function, to presympathetic neurons residing in RVLM contributed to sympathetic overactivity and increased respiratory modulation of BP (Moraes et al., 2014). In human studies, increased chemoreflex sensitivity was reported in patients with established hypertension and borderline hypertension, and also in normotensive subjects with a family history of hypertension (Tafil-Klawe et al., 1985; Somers et al., 1988; Paton et al., 2013). This augmentation of chemoreceptor sensitivity could lead to an enhanced respiratory-sympathetic coupling, and thus may cause an increase in the magnitude of respiratory-related variations in BP. Apart from chemoreflex function, other potential mechanisms which may promote the larger SBP variation mediated by respiration in hypertensive patients, such as changes in peripheral sympathetic property, vascular response, endothelial dysfunction, and other BP regulatory mechanisms, remain to be elucidated in future studies.

### Respiratory Modulation at MAT

As expected, MAT elicited significant changes in homeostatic control of the cardiovascular and respiratory systems (Table 2 and Figure 1). Changes in cardiovascular parameters including significant increases in HR, BP, and CO (Yoshiuchi et al., 1997), and consistent with previous studies, the increments were comparable between hypertensives and normotensives (Lindqvist et al., 1993). The responses of ventilation to psychological stress were usually increases in respiratory frequency and respiratory instability in human studies (Wilhelm et al., 2001; Tipton et al., 2017). In line with this, respiratory rate and the variabilities of expiratory time and respiratory rate were significantly increased in normotensives during MAT, and our data indicated that the increased respiratory rate was largely attributed to the decreased inspiratory time. A potentially intriguing observation in the present study is that in hypertensive patients, the inspiratory time was decreased as similar to normotensives, whereas the expiratory time tended to increase during MAT (2.23 ± 0.14 vs. 2.55 ± 0.23 s, *P* = 0.067), and as a result respiratory rate did not significantly increase during MAT in hypertensive patients. From the analysis made in the present study, we cannot make meaningful interpretations about how this difference between groups might occur. One explanation is that variation in ventilatory response of individuals to mental stress is associated with cognitive function (Wilhelm et al., 2001; Tipton et al., 2017), which was reported to be impaired in hypertensive patients (Paglieri et al., 2008; Elias et al., 2012).

Although MAT elicited significant changes in respiratory and cardiovascular parameters, our data showed that magnitudes of the respiratory regulation on HR and BP remained unchanged. The respiratory modulation indexes calculated by event-triggered averaging method were not changed during MAT, except the Norm_ΔSBP (Figure 2). Considering that the ΔSBP did not change significantly, the decrease in the Norm_ΔSBP was most probably due to the elevated SBP during MAT (Norm_ΔSBP = ΔSBP/maximum of SBP). The percentages of powers of respiratory-related variations in cardiovascular signals were also not changed during MAT, and no significant main effect of conditions on temporal respiratory regulation of HR and BP. Collectively, our data revealed that MAT did not influence the respiratory-related variations in HR and BP. There are both mathematical and physiological considerations for this observation. First, a limitation of the event-triggered averaging method in accessing respiratory modulation is that it is only sensitive to the phase locked responses to the triggering events. Averaging across epochs could attenuate other variations in the cardiovascular signals and preserve the respiratory-related components. However, the respiratory-related components specifically refer to activities that are phase-locked to respiration. In other words, if there were phase shifts in the responses of cardiovascular variables to respiration during MAT, these responses will be canceled out during the averaging process. Second, the intimate relationship between the cardiovascular and respiratory systems is resulted from the central coupling of respiratory networks to cardiovascular neural circuits in the brainstem and modulatory feedbacks from cardiorespiratory afferents (Shantsila et al., 2015). This coupling between respiratory and cardiovascular regulation could be disrupted during mental stress because psychological stress activates cortical brain regions, which interact with various subcortical brain regions and generate cardiorespiratory responses through a “top-down” mechanism (Gianaros and Wager, 2015). This central command influences both central respiratory and cardiovascular networks and produces highly coordinated respiratory and cardiovascular responses (Dampney, 2015). In this regard, the central mechanism may act as a “confounding variable” and produce a spurious association between the cardiovascular and respiratory systems, causing unchanged respiratory variations in cardiovascular signals during MAT in this study. Unfortunately, the methods used in the present study are not able to disentangle these mechanisms, and future investigations are needed to elucidate the mechanism underlying the cardiorespiratory responses to mental stress.

### Clinical Perspectives

In a recent large population study, BP variability was significantly associated with increased risk of adverse cardiovascular outcomes in hypertensive patients, and a 5 mmHg increase in the within-visit standard deviation of SBP could increase the risk of cardiovascular events by 15% (Mehlum et al., 2018). We found a comparable value of absolute respiratory-related variation in SBP (4.41 ± 0.27 mmHg) at rest in hypertensive patients in this study. Additionally, young adults with a high risk of developing hypertension also showed an exaggerated respiratory modulation of SBP (Menuet et al., 2017). Considering these findings, future studies assess the long-term prognostic value of respiratory modulation of SBP in hypertensive patients as well as normotensives are of great interest. If the prognostic importance is confirmed in larger population studies, the assessment of this respiratory-related variation in SBP in hypertensive patients and therapeutic methods to alleviate this variability may be recommended. Previous studies have proposed manipulating respiratory pattern by device-guided or yogic practice as non-pharmacological interventions to lower BP (Joseph et al., 2005; Sengupta, 2012). However, latter studies did not support a long-term efficacy of breathing training for hypertension (Landman et al., 2014). Our study found that the magnitude of respiratory modulation of BP was not influenced by the changed respiratory pattern induced by mental stress. Therefore, our data indicated the need of new treatment strategies to attenuate the BP variability related to respiration. Given that several underlying cardiorespiratory pathologies of this exaggerated respiratory modulation have been established in animal studies (Moraes et al., 2014; Menuet et al., 2017), pharmacological treatment targeting these mechanisms could be considered.

### Limitations

There are several limitations. First, as discussed, the event-triggered averaging method could not detect non-phase-locked responses of cardiovascular variables to respiratory regulation. Second, although orthogonal subspace projection could take non-stationarity of the signal into account by including wavelets as a basis when constructing the subspace of respiratory signal, this method only considers the linear relationship between cardiorespiratory signals. The non-linear characteristics of cardiorespiratory interrelationship should be further studied. Third, the present study was a case-controlled study with small sample size. Future multicenter population studies are needed.

## Conclusion

The present study demonstrated that respiratory modulation of SBP was exaggerated in patients with essential hypertension. Furthermore, this modulation remained unchanged despite significant changes in respiration and cardiovascular variables during mental stress. The larger SBP variation mediated by respiration in hypertensive patients may contribute to the heightened risk of adverse cardiovascular consequences. The prognostic relevance of respiratory modulation of BP, and whether it should be recommended as a BP control target are questions that warrant investigations in future studies.

## Data Availability

The datasets generated for this study are available on request to the corresponding author.

## Ethics statement

The studies involving human participants were reviewed and approved by Ethical Committee of Xi’an Jiaotong University. The patients/participants provided their written informed consent to participate in this study.

## Author Contributions

LX and JZ designed the study. LX, XD, and FZ performed the experiments. LX, XD, JY, BL, and XW processed the data and designed the figures. LX, JZ, ZL, and CL interpreted the data and drafted the manuscript. All authors discussed the results and contributed to the final manuscript.

## Conflict of Interest Statement

The authors declare that the research was conducted in the absence of any commercial or financial relationships that could be construed as a potential conflict of interest.
